# Senescence, Stress, and Reactive Oxygen Species

**DOI:** 10.3390/plants4030393

**Published:** 2015-07-08

**Authors:** Ivan Jajic, Tadeusz Sarna, Kazimierz Strzalka

**Affiliations:** 1 Department of Plant Physiology and Biochemistry, Faculty of Biochemistry, Biophysics and Biotechnology, Jagiellonian University in Krakow, Gronostajowa 7, Krakow 30-387, Poland; E-Mail: ivan.jajic1705@gmail.com; 2 Department of Biophysics, Faculty of Biochemistry, Biophysics and Biotechnology, Jagiellonian University in Krakow, Gronostajowa 7, Krakow 30-387, Poland; E-Mail: tadeusz.sarna@uj.edu.pl; 3 Malopolska Centre of Biotechnology, Jagiellonian University in Krakow, Gronostajowa 7, Krakow 30-387, Poland

**Keywords:** leaf senescence, abiotic stresses, reactive oxygen species, hydrogen peroxide, singlet oxygen, superoxide anion

## Abstract

Generation of reactive oxygen species (ROS) is one of the earliest responses of plant cells to various biotic and abiotic stresses. ROS are capable of inducing cellular damage by oxidation of proteins, inactivation of enzymes, alterations in the gene expression, and decomposition of biomembranes. On the other hand, they also have a signaling role and changes in production of ROS can act as signals that change the transcription of genes that favor the acclimation of plants to abiotic stresses. Among the ROS, it is believed that H_2_O_2_ causes the largest changes in the levels of gene expression in plants. A wide range of plant responses has been found to be triggered by H_2_O_2_ such as acclimation to drought, photooxidative stress, and induction of senescence. Our knowledge on signaling roles of singlet oxygen (^1^O_2_) has been limited by its short lifetime, but recent experiments with a *flu* mutant demonstrated that singlet oxygen does not act primarily as a toxin but rather as a signal that activates several stress-response pathways. In this review we summarize the latest progress on the signaling roles of ROS during senescence and abiotic stresses and we give a short overview of the methods that can be used for their assessment.

## 1. Introduction

Senescence in plants is a complex deterioration process that can lead to the death of whole organisms or a single organ. It is regulated by autonomous (internal) factors (age, reproductive development, and phytohormone levels) and by environmental signals, including photoperiod, stresses such as drought, ozone, nutrient deficiency, wounding, and shading [1]. The generation of reactive oxygen species (ROS) is one of the earliest responses of plant cells under abiotic stresses and senescence [2,3]. In plants, ROS are formed as byproducts of aerobic energy metabolism and of plants being exposed to various biotic and abiotic stresses [4,5,6]. Under normal conditions, the production of ROS in cells is maintained at low levels by antioxidant enzymes. This balance can be disrupted by a depletion of antioxidants or the excess accumulation of ROS, leading to oxidative stress, and consequently to damage to cellular macromolecules and membranes and an increase in lipid peroxidation [7,8]. ROS-induced oxidative stress limits agricultural yields worldwide [9]; in the United States alone, it is estimated that the deleterious effects of abiotic stresses on agricultural production are responsible for losses amounting to billions of dollars annually [10]. Plants have evolved different mechanisms to protect themselves from adverse environmental conditions, such as the process of acclimation, which involves less ROS production coupled with an efficient antioxidant defense [11,12] and the activation of different signaling pathways [13,14].

Initially, ROS were exclusively considered toxic metabolic products that can damage cellular components, but now it is clear that ROS play a dual role in plants both as toxic compounds and as key regulators of many biological processes [15,16,17]. The important role of ROS in signaling has been demonstrated in many studies. It has been shown that ROS modulate the activity of key signaling compounds such as MAP kinases [18], provide protection against pathogen invasion [19,20], stimulate abiotic stress tolerance [21], and have an important role during early responses to wounding [22]. Despite the importance of ROS, our knowledge of the mechanism of their action is still limited. In this review we will try to summarize the latest progress on the roles of reactive oxygen species during senescence and abiotic stresses.

## 2. ROS Detection 

The main problem with an accurate determination of the role of ROS in senescence and abiotic stresses is the simultaneous generation of ROS [23,24] and the limited number of non-invasive and specific methods that can be used for their determination. For example, in plants suffering from moderate light stress singlet oxygen (^1^O_2_), superoxide anion (O**^•^**_2_**^−^**) and hydrogen peroxide (H_2_O_2_) are released simultaneously [23], making it difficult to establish their individual roles. This is further complicated by significant differences in the production of ROS when senescence is induced artificially and when the plant ages naturally [25]. The effects of H_2_O_2_ on gene expression have also been reported to be different when the H_2_O_2_ was applied exogenously and when it was induced in response to high light [26]. Finally, several studies revealed that multiple stressors, as usually encountered by plants in nature, could substantially change the expression patterns of genes determined in a single factor analysis [27,28,29,30]. There is a need for an experimental model that can take into account all the factors that can influence the outcome of research into the role of ROS.

There are a great number of user-friendly assays that can be used for the measurement of ROS production in plant tissues, including the fluorometric determination of H_2_O_2_ with Amplex Red [31] and the colorimetric determination of O^•^_2_^−^ with XTT [32]. Although easy to use, these methods lack specificity. This is because Amplex Red is somewhat unstable—it can be autooxidized and produce O^•^_2_^−^ and H_2_O_2_ [33], and it can react not only with H_2_O_2_ but also with other redox active compounds [34], while XTT can be reduced by short-chain sugars [35]. Higher specificity can be achieved by using the spin trapping technique by electron paramagnetic resonance (EPR) spectroscopy. Spin trapping involves the reaction between a nitrone or nitroso compound and a free radical to form a stable spin adduct [36]. The spin adduct usually yields a distinctive EPR spectrum characteristic of the particular free radical that is trapped. Under normal conditions, the flux of ROS generated in cells is maintained at low levels by the action of antioxidant enzymes and low molecular weight antioxidants, making the detection of ROS difficult. In order to successfully detect ROS, an imbalance between the production of ROS and their decay needs to be created. This can be achieved by the illumination of samples with visible light in the presence of an appropriate spin trap or with the addition of the spin trap immediately after illumination [37,38,39,40]. There are numerous publications with detailed descriptions of how to use EPR spectroscopy for the detection of hydrogen peroxide [39], the superoxide anion [40], the hydroxyl radical [41], and singlet oxygen [38] in plant tissues. One of the limitations of spin trapping is that the method does not provide information on the specific sites of ROS production in tissues due to solvent incompatibility with living tissue and high concentrations of spin traps needed [42], Also, it typically underestimates actual *in situ* ROS concentrations because only a small fraction of the radicals produced is usually trapped [43]. Non-invasive, *in vivo* measurement of ROS can be achieved using fluorescent probes in combination with confocal laser scanning microscopy (CLSM) [44] or fluorescence microscopy [45,46,47]. One advantage of CLSM methodology is the possibility of studying the intracellular location of ROS using simultaneously specific fluorescent probes for different organelles. 2′,7′-Dichlorofluorescein diacetate (DCF-DA) can be used for monitoring H_2_O_2_ in plant tissues [48]; however, it lacks specificity since it was demonstrated that it can also react with other peroxides [49]. Dihydroethidium (DHE) is a more specific probe that can be used for detection of O^•^_2_^−^ in different plant tissues [50,51]. The determination of singlet oxygen with a singlet oxygen sensor green (SOSG) reagent showed high specificity to ^1^O_2_ without the interference of hydroxyl radicals or superoxide [46]. Unfortunately, even in the absence of external ^1^O_2_ photosensitisers, the indicator can be converted to its green fluorescent form upon exposure to either UV or visible radiation. This could obviously lead to a wrong estimation of ^1^O_2_ levels [52]. Real-time monitoring of reactive oxygen species in living cells and tissues can be achieved with a genetically encoded redox probe such as HyPer and reduction-oxidation sensitive green fluorescent protein (roGFPs) [53]. These probes offer high specificity and can be used for determination of ROS in various subcellular compartments. HyPer is highly sensitive to hydrogen peroxide, is insensitive to other oxidants, and it does not cause artifactual ROS generation, thus having great potential in plant biology [54], while roGFP can be used for determination of H_2_O_2_, but also for determination of thiol redox state depending on its form [53]. Disadvantages of these probes include the necessity of pH control and possible antioxidant activity, which need to be taken into consideration [53]. In Table 1 we summarize the methods that can be used for measurement of ROS production in plant samples.

**Table 1:** Overview of the methods for determination of ROS in plan samples.

Method/Probe	Advantage	Disadvantage	Used for
Amplex Red	Easy to use and fast	Can be autooxidized, reacts with other redox reactive compounds	H_2_O_2_
XTT	Easy to use and fast	Non-specific, can be reduced by short-chain sugars	O^•^_2_^−^
EPR spin trapping	Highly specific, can be used for determination of numerous ROS	Expensive, laborious, method does not provide information on the specific sites of ROS production in tissues	H_2_O_2_, O^•^_2_^−^, ^1^O_2_, OH^−^
DCF – DA	*In vivo* determination of intracellular ROS production	Non-specific, reacts with other peroxides, expensive equipment	H_2_O_2_
DHE	*In vivo* determination of intracellular ROS production	Expensive equipment	O^•^_2_^−^
SOSG	*In vivo* determination of intracellular ROS production	Wrong estimation of ^1^O_2_ upon exposure to visible or UV light	^1^O_2_
HyPer and roGFP	Real time monitoring of ROS in living cells and tissues	Necessity for pH control and possible antioxidant activity	H_2_O_2_

## 3. Superoxide Anion 

In plants O^•^_2_^−^ is generated in different cell compartments, including chloroplasts, peroxisomes, apoplast, the mitochondrial electron transport chain, and the plasma membrane [55,56,57]. The primary source of the superoxide anion in chloroplasts are Mehler reactions, during which O_2_ is reduced by electrons from the photosynthetic electron transport chain [58]. Generated O**^•^**_2_**^−^** is then converted to hydrogen peroxide (H_2_O_2_), mostly by the action of CuZn-superoxide dismutase (SOD) [59]. Thus, SOD determines the lifetime of O**^•^**_2_**^−^** in cells and the probability of its involvement in biochemical processes. Furthermore, O^•^_2_^−^ is a moderately reactive, short-lived ROS with a half-life of approximately 2–4 µs [17], and it cannot cross the chloroplast membrane [60]. For the reasons listed above, a signaling role of O**^•^**_2_**^−^** during senescence and abiotic stresses seems unconvincing. However, chloroplasts are not the only sites of O**^•^**_2_**^−^** production. In peroxisomes, O^•^_2_^−^ is being generated by two different sources: in peroxisomal matrix via action of enzyme xanthine oxidase [61,62] and by electron transport chain (ETC) in peroxisomal membrane [63]. Peroxisomes can be considered as an important source of signaling molecules since they have capacity to rapidly produce and scavenge H_2_O_2_ and O^•^_2_^−^ due to the presence of many antioxidants in these organelles. Another important source of O^•^_2_^−^ in plant cells are NADPH oxidases (NOX), in plants commonly known as respiratory burst oxidase homologs (Rbohs), which catalyze the production of O^•^_2_^−^ [64,65]. Plant Rbohs have been intensively studied recently since they play key roles in many physiological processes, such as ROS signaling and stress responses [66,67]. Finally, O**^•^**_2_**^−^** is also produced in cytosol by action of xanthine dehydrogenase and the aldehyde oxidase [68,69]. Numerous studies have reported an increase in the production of O**^•^**_2_**^−^** during natural and artificially induced senescence [70,71]; however, attributing a specific signaling role to this increase is extremely difficult since the increase in most cases is accompanied by the production of other ROS and the quick conversion of O**^•^**_2_**^−^** to H_2_O_2_. High production of ROS is damaging to the cell due to oxidative modifications of key cellular components and may ultimately lead to plant cell death [72]. In a recent study [73] it was shown that under the high temperature treatments, large amounts of O^•^_2_**^−^** and H_2_O_2_ were generated and accumulated in cucumber leaves, leading to premature senescence, which is indicated by the changes in protein, lipid peroxidation (LPO), and chlorophyll content. Nevertheless, a signaling role of O^•^_2_**^−^** was demonstrated in *Arabidopsis thaliana* plants exposed to methyl viologen, a superoxide anion propagator, under light. The generation of O^•^_2_**^−^** in the absence of H_2_O_2_ accumulation revealed a subset of nuclear encoded genes that are likely to be specific for an O^•^_2_**^−^**-mediated signaling pathway [74]. Data analysis identified a strong upregulation of genes belonging to categories functioning in abiotic stress responses, among them WRKY6, which has previously been reported to play a role during senescence and in defense-related processes [75]. Recently, we investigated the production of O^•^_2_**^−^** during the development and senescence of secondary barley leaves by using EPR––a spin trapping method with DMPO as a spin trap [76]. It was shown that the production of O^•^_2_**^−^** increases during the development of barley, reaching its highest level right after the onset of senescence. Thereafter, the production of O^•^_2_**^−^** started to decline till the end of the senescence process. This was accompanied with an increase in membrane fluidity during the same period [77], which could be a factor facilitating the increase in the generation of ROS. An increase in O^•^_2_**^−^** was also observed in the interveinal area of senescing tobacco leaves, as well as in the minor veins of mature and senescent leaves, while it was absent in the major veins [78]. It is hypothesized that spatial differences in the superoxide anion are important for the non-uniform downregulation of photosynthesis-associated genes. A further role of O^•^_2_**^−^** as a signaling molecule was demonstrated during the early wound response in an experiment with *Medicago* leaves where ROS production was inhibited with diphenyleneiodonium (DPI). The rapid (≤3 min) DPI inhibition of phase I O^•^_2_**^−^** production suppressed the differential regulation of 7 out of 19 wound responsive proteins, showing that early, wound-related O^•^_2_**^−^** production (phase I) provides an essential signal for wound-related changes in the leaf apoplast proteome [22]. Increased production of O^•^_2_**^−^** was observed in plant responses to cadmium stress in pea (*Pisum sativum* L.) [48]. Exposure to Cd leads to an oxidative stress as a result of disturbance in antioxidant defense and a decrease in NO level. It was demonstrated that NO can mitigate the deleterious effect of Cd on lupine roots [79] and that it has a possible antioxidant effect in its ability to react with O^•^_2_**^−^** to prevent oxidative damage [80]. In this way, O^•^_2_**^−^** could contribute to plant responses to abiotic stresses.

## 4. Hydrogen Peroxide

**Figure 1:**
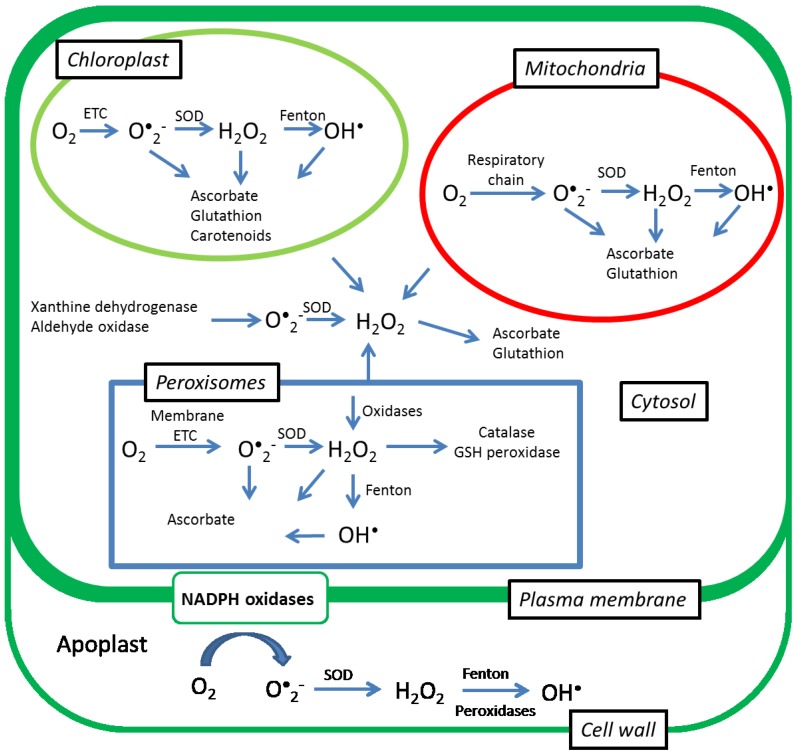
Production and scavenging of ROS in a plant cell. Figure legend: Superoxide dismutase (SOD); Fenton (decomposition of hydrogen peroxide to highly reactive hydroxyl radical in presence of iron); ETC (electron transport chain).

Hydrogen peroxide plays an important role in plants under stress conditions as a signaling molecule that mediates between different physiological processes [81]. It is involved in the regulation of the senescence process [82], protection against pathogen attack [83], the reduction of stress intensity at low light [84], and the alleviation of drought stress [85], and it can influence the expression of hundreds of genes [86]. Hydrogen peroxide is produced in plants via two possible pathways: dismutation of O^•^_2_**^−^** with the involvement of SOD [59], and via oxidases such as amino and oxalate oxidases [87]. The level of H_2_O_2_ is kept under control by a fine-tuned network of enzymatic and low-molecular-weight antioxidants that prevent the excess accumulation of H_2_O_2_ [88]. Production and scavenging of H_2_O_2_ in plant cells has been summarized in Figure 1. The balance between SODs and the different H_2_O_2_-scavenging enzymes in cells is considered to be crucial in determining the steady-state level of H_2_O_2_ [89]. In comparison with other ROS, H_2_O_2_ is the most stable and least reactive ROS, and it can easily cross the membrane [81,90], which makes it a good signaling molecule. H_2_O_2_ plays a versatile role in plants; as a signaling molecule it is involved in the regulation of various abiotic and biotic stresses [81] and, at high concentrations, it has an important role in cell death and during the final stages of senescence, when it contributes to cell degradation [16,17]. The dual role of H_2_O_2_ was confirmed in a recent study, in which treatment with 600 µM H_2_O_2_ caused an increase in the vase life of a cut Oriental × Trumpet hybrid lily “Manissa,” while concentrations of 800 and 1200 µM resulted in negative effects [91]. Further evidence that the effects of H_2_O_2_ are dose dependent comes from a study in which wax apple trees were spray-treated with different concentrations of H_2_O_2_ under field conditions [92]. Spraying wax apple fruits with 5 and 20 mM of H_2_O_2_ once a week produced better fruit growth and maximized the yield and quality in comparison with the control and with a higher dose of 50 mM of H_2_O_2_.

H_2_O_2_ plays an important role during the senescence process, where it was shown that it could be used as a signal to promote senescence in different plant species, and to be part of a complex regulatory network [93]. It was shown that H_2_O_2_ increases at the point when the plants start to bolt and flower, which is supported by a decrease in ascorbate peroxidase 1 activity at the same time [16]. This probably induces the expression of transcription factors and senescence-associated genes. Indeed, expression analysis showed that H_2_O_2_ treatment induced 14 out of 36 salt-triggered senescence-associated genes and 15 senescence-associated NAC genes [94], indicating that salt-triggered senescence at least in part involves H_2_O_2_-mediated signaling through NAC transcription factors. This is supported by studies in which it was demonstrated that H_2_O_2_ treatment induces the expression of NAC transcription factors ORS1 [95] JUB1 [96] and ATAF1 [97], which have a significant impact on progression of senescence. Overexpression of ORS1 triggers the expression of senescence-associated genes and accelerates senescence in transgenic plants, whereas its inhibition delays it. Contrary to ORS1, the overexpression of JUB1 strongly delays senescence, dampens intracellular H_2_O_2_ levels, and enhances tolerance to various abiotic stresses. *ATAF1* overexpression stimulates the progression of senescence by activating senescence promoting transcription factor *ORE1* and repressing chloroplast maintenance transcription factor *GLK1*. The generation of H_2_O_2_ during the development and senescence of barley was investigated in our recent study [76]. The results showed an increase in the production of H_2_O_2_ with the development of barley, with the highest levels observed right after the induction of senescence and at the very end of the senescence process, which is similar to the pattern observed in the study discussed above [16]. Our research provides further evidence of the important role of H_2_O_2_ during leaf senescence in two different aspects: as a signal molecule for the induction of senescence, and in the degradation of molecules at later stages of senescence.

Several studies have indicated that H_2_O_2_ can interplay with other signal molecules that are important for plant development and during senescence such as abscisic acid (ABA) and ethylene [12,98]. It was shown that H_2_O_2_ could be involved in the signaling of plant growth regulators such as ethephon [98]. The application of ethephon results in an elevation in H_2_O_2_ levels, which is accompanied by the increased expression of sweet potato catalase. The elimination of H_2_O_2_ influence by exogenous-reduced glutathione alleviates ethephon-mediated effects. Recently, the interaction between ABA, H_2_O_2_, and ascorbic acid in Mediterranean shrubs during summer drought was investigated. It was shown that the drought stress–ABA–H_2_O_2_ interaction can induce an increase in ascorbic acid, maintaining and even decreasing the ascorbate oxidative status under summer drought conditions, thereby protecting plants from oxidative damage.

There are numerous studies demonstrating the important role of H_2_O_2_ in the acquisition of tolerance to different abiotic and biotic stresses [84,85,99,100]. It was recently shown that pre-treatment with H_2_O_2_ provides protection against heat stress and low light induced oxidative stress by modulating the activity of antioxidant enzymes. The exogenous application of H_2_O_2_ can induce tolerance to heat stress in seedlings of *Cucumis sativus* cv Lvfeng no. 6 [100]. The pre-treatment of cucumber leaves with H_2_O_2_ and heat increased antioxidant enzyme activities, decreased lipid peroxidation, and thus protected the ultrastructure of chloroplasts under heat stress. Similarly, it has been shown that exogenous H_2_O_2_ can have a beneficial effect on low light induced oxidative stress [84]. Low light induces an oxidative stress [101], which increases ROS and causes lipid peroxidation. H_2_O_2_ pre-treatment of cucumber leaves resulted in decreased levels of O^•^_2_**^−^**, endogenous H_2_O_2_, and malonaldehyde by moderating the activities of antioxidant enzymes, thus reducing lipid peroxidation and stress intensity at low light. Pre-treatment with H_2_O_2_ can also increase drought stress tolerance in soybean leaves by promoting the expression of stress-response genes [85]. Exogenous application of H_2_O_2_ caused an increase in the mRNA levels of key enzymes for the biosynthesis of oligosaccharides, which are known to help plants tolerate drought stress. This enabled the soybean plant to avoid drought stress through the maintenance of leaf water content and thus to delay foliar wilting. Finally, hydrogen peroxide contributes to defense responses against pathogens. It was demonstrated that H_2_O_2_ is important for the greater tolerance of kumquat leaves infected with *Xanthomonas axonopodis* than that of grapefruit [19]. Infected kumquat leaves have a high accumulation of H_2_O_2_, which is promoted by the suppression of ascorbate peroxidase activity and later by the suppression of catalase activity, both involved in maintaining H_2_O_2_ at low levels. H_2_O_2_ can then be used as a substrate for the higher activity of Class III peroxidase in the apoplast, which is known to be involved in plant defense against pathogens.

## 5. Singlet Oxygen

Singlet oxygen is the highly reactive, excited state of molecular oxygen that can be formed in a reaction between O_2_ and the chlorophyll triplet state [102]. Unlike the formation of H_2_O_2_ and O^•^_2_**^−^**, the formation of ^1^O_2_ is not accompanied by the transfer of an electron to O_2_. Instead, one of the unpaired electrons is promoted to a higher energy orbital [103]. Under normal conditions, ^1^O_2_ is generated during photosynthesis by the photo activation of photosensitizers, mainly chlorophylls and their precursors [102]. Singlet oxygen is also generated during senescence [25] and under different abiotic stresses [104,105,106]. Similarly to other ROS, ^1^O_2_ has a dual effect. As an oxidizing agent it can react with various biological molecules, causing damage and leading to cell death [107]. It can also play a signaling role by activating the expression of different genes [23,108]. As a result of its high reactivity and short lifetime of 3.1 to 3.9 µs in pure water [109], ^1^O_2_ is able to interact with molecules mostly in its nearest environment. The diffusion distance of ^1^O_2_ has been calculated to be up to 10 nm in a physiologically relevant situation [110]. On the other hand, it was demonstrated that ^1^O_2_ is capable of diffusing a distance of over 270 nm in rat nerve cells [111] and that ^1^O_2_ produced in the photosynthetic apparatus of *C. reinhardtii* under high light is capable of leaving the thylakoid membrane and reaching the cytoplasm or even the nucleus [112], which makes its role as a signaling molecule feasible.

Significant progress in the investigation of the role of singlet oxygen in signaling was achieved with the use of a conditional fluorescent (*flu*) mutant of *Arabidopsis* that accumulates the photosensitizer protochlorophyllide in the dark and generates singlet oxygen after transfer to light [107,113]. Following illumination with light, a different set of nuclear genes are activated within the *flu* mutant, and they are different from those induced by O^•^_2_**^−^** and/or H_2_O_2_, suggesting that singlet oxygen does not act primarily as a toxin but rather as a signal that activates several stress-response pathways [107]. Recently, it was reported that ^1^O_2_ could be responsible for increased tolerance to photooxidative stress in *Arabidopsis* plants through the action of β-cyclocitral [114]. β-cyclocitral is a β-carotene derivative produced in high light that is able to induce changes in the expression of a large set of genes, which strongly overlap with the network of genes induced by ^1^O_2_ [114]. At the same time it has little effect on the expression of H_2_O_2_ gene markers. β-cyclocitral-induced reprogramming of gene expression is associated with increased tolerance to photooxidative stress, indicating that β-cyclocitral is a stress signal produced in high light that is able to induce defense mechanisms and represents a likely messenger involved in the ^1^O_2_ signaling pathway in plants [114]. Further evidence that ^1^O_2_ participates in acclimation to photooxidative stress comes from a study with an npq1lut2 double mutant [106]. The npq1lut2 mutant specifically accumulates ^1^O_2_ due to its selective loss of lutein and zeaxanthin, which participate in the quenching and scavenging of ^3^Chl* and ^1^O_2_ [115,116]. Following high light illumination, ^1^O_2_ accumulates and modifies the expression of a group of genes encoding chloroplast proteins, leading to a significant change in chloroplast composition and functional modifications. High light induced ^1^O_2_ responses were also investigated in an *Arabidopsis* cell suspension culture (ACSC) containing functional chloroplast [117]. An experiment with different fluorescent probes showed that the high light treated cultures emitted fluorescence that corresponded with the production of ^1^O_2_. This was accompanied by significant changes in the expression of transcripts specifically upregulated by ^1^O_2_, which leads us to conclude that ^1^O_2_ plays an important role in the initiation of defense responses to high light.

**Figure 2:**
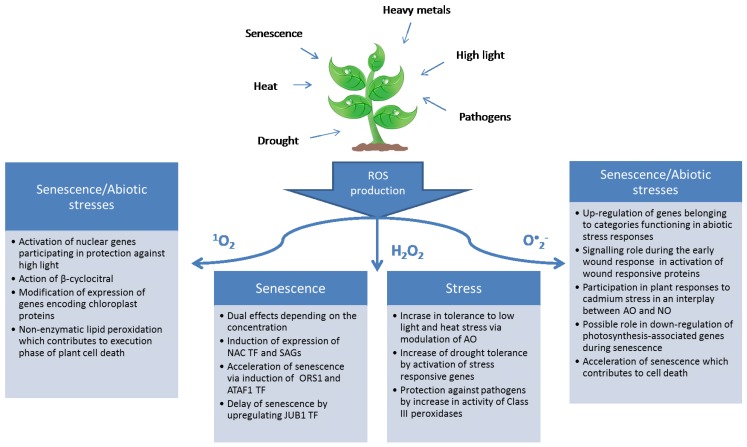
Possible roles of ROS during senescence and abiotic stresses.

When it comes to senescence, our knowledge on the signaling role of the ^1^O_2_ is limited by the scarcity of the research relevant to the topic. There is general agreement that ROS production increases during senescence [2,3]. However, the increase in ^1^O_2_ is observed simultaneously with that of other ROS, making it difficult to isolate the role of ^1^O_2_. It was reported that ^1^O_2_ is the main cause of senescence-associated oxidative stress in chloroplasts of sage [118]. However, this was concluded on the basis of the strong degradation of β-carotene and α-tocopherol in drought-stressed plants, which suggests the enhanced formation of singlet oxygen. In addition, in a recent study, a mass generation of singlet oxygen was measured in the early stages of hormone-treated barley but then declined, while in naturally senescing plants there was continuous production of low amounts of singlet oxygen [25]. Together with an increase in ^1^O_2_, artificially senescing plants contained oxidative breakdown products of β-carotene such as β-cyclocistral, which is a possible messenger involved in the ^1^O_2_ signaling pathway in plants [114], while the same was undetectable in a naturally senescing plant. Similar results were observed in our recent study, where it was shown that during the development and senescence of secondary barley leaves there is a continuous production of low amounts of ^1^O_2_ [76]. Another possible role of ^1^O_2_ could be its contribution to an increase in lipid peroxidation (LPO), leading to cell death. It is well known that with advancing senescence there is a notable increase in LPO [119,120]. Recently, it has been reported that in optimal growth conditions ^1^O_2_ was responsible for more than 80% of the non-enzymatic LPO in *Arabidopsis* leaf tissues [121]. Lipid peroxidation leads to the generation of free radicals, which can lead to the promotion of senescence [122]. This in turn leads to an increase in lipoxygenase activity, which can further increase LPO and also form ^1^O_2_ [123], leading to overproduction of ^1^O_2_. Indeed, it was showed that in *Arabidopsis* mutants favoring ^1^O_2_ production, photooxidative stress led to a dramatic increase in LPO preceding cell death [121]. Possible roles of ROS during senescence and stress are summarized in Figure 2.

## 6. Conclusions

In this review we have given a short overview on the possible role of three classes of ROS during senescence and abiotic stresses. ROS play an important role in different plant processes ranging from plant stress adaptation to defense against pathogen attack. In the ROS family, the signaling roles of H_2_O_2_ have been most thoroughly studied due to its relative stability and ability to diffuse through membranes. It has been demonstrated that H_2_O_2_ plays various roles in plant growth, development, and metabolism. It has an important role during the senescence process, where it was shown that it could be used as a signal to promote senescence and during the acquisition of tolerance to different abiotic and biotic stresses. In comparison with H_2_O_2_, the superoxide anion is less stable and cannot cross the membrane, which makes it less suitable as a signal molecule. Nevertheless, an important signaling role of O^•^_2_**^−^** was demonstrated when it was shown that the generation of O^•^_2_**^−^** in the absence of H_2_O_2_ leads to a strong upregulation of the genes that function in abiotic stress responses and during senescence. Finally, in recent years, with the discovery of a *flu* mutant, it was demonstrated that ^1^O_2_ is capable of activating a set of nuclear genes different from those activated by O^•^_2_**^−^** and H_2_O_2_ and that it plays an important role in plant responses to light.

At present, only the role of H_2_O_2_ during senescence and abiotic stresses has been extensively studied, while the role of other ROS remains to be further clarified. In recent years there has been significant progress in this area, with development of new techniques and technologies, but still there is no ideal technique that can be applied to a variety of systems and to specific ROS classes. EPR spectrometry can be used to measure specific ROS species but it requires a thorough sample preparation, which prevents the measurement of specific sites of ROS production in tissues. On the other hand, genetically-encoded redox probes can be used to measure ROS production in different cell compartments *in vivo* but they often do not differentiate between different classes of ROS. Moreover, most of the papers investigate the impact of a group of ROS, while the contribution of individual ROS remains questionable. Significant progress in the future can be made on the signaling roles of O^•^_2_**^−^** and ^1^O_2_ during senescence. Roles of these two classes during this important process have not yet been sufficiently investigated and many questions wait to be answered. Already it has been shown that O^•^_2_**^−^** upregulates some of the genes that are important during senescence such as WRKY6, but not much more is known. Similarly, the role of ^1^O_2_ during senescence is limited to its contribution to cell death, while several studies reported that production of this species remains unchanged during natural senescence. Whether this is true or not remains to be seen.
